# Digital Humanitarianism: Collective Intelligence Emerging

**DOI:** 10.1371/journal.pmed.1001278

**Published:** 2012-07-31

**Authors:** The PLoS Medicine Editors

## Abstract

The *PLoS Medicine* editors discuss the concept of Digital Humanitarianism and the possibilities it affords for facilitating information in crises and the delivery of relief and development aid.

Last month at the Médecins Sans Frontières (MSF) Scientific day (http://www.msf.org.uk/Scientific_Day.aspx), a session on digital humanitarianism, with a keynote given by Paul Conneally from the International Telecommunication Union (ITU), offered a vision of a future where huge amounts of data can be collected, analyzed, and used to facilitate the delivery of relief and development aid and information in some of the most challenging settings on earth. The talk acknowledged there are obstacles to be overcome—such as the management of privacy issues—but the overall vision was a compelling one that clearly resonated with its audience.

This is a fast-moving field, and it's not entirely clear what “digital humanitarianism” fully encompasses, especially as every new emergency seems to trigger some new innovation. Perhaps not surprisingly, much of the thinking, discussion, and dissemination of ideas in this area is going on in blogs (e.g., http://irevolution.net/, http://blog.standbytaskforce.com/, http://blogs.plos.org/speakingofmedicine/2012/03/21/geeks-and-primitive-fieldworkers-a-tale-of-two-cultures/) and Twitter postings (e.g., @andrejverity, @PatrickMeier, @conneally, @ushahidi). However, as the field develops, key questions are emerging such as what data and metadata should be collected; how these data can be managed meaningfully and safely; what tools and expertise are needed to synthesize these data; how to coordinate the many related initiatives; whether there are lessons to be learned from related initiatives; and, a bit closer to home, what role journals might play in this.

Digital humanitarianism is currently best illustrated by two examples. First, following the 2010 earthquake in Haiti, mobile phones were used both in an expected way (though not at the volumes expected) by individuals calling, texting, and using social media for aid, and in an unexpected way, to track population movements after the earthquake [1]. Second, worldwide, thousands of remote volunteers have aided in tracking and mapping humanitarian needs online. This mapping also occurred following the Christchurch earthquake and more recently has been used in political crises such as that in Libya.

These examples share several key characteristics. First, they demonstrate the sheer volume of data available and in times of crisis how quickly the data can be compiled, analyzed, and used. Such volumes of data mean that the techniques that are often used in emergencies to synthesize the data collected will need to be rethought. Even though many organizations—such as UNICEF, Office for the Coordination of Humanitarian Affairs, World Food Programme, UN High Commission for Refugees, WHO, MSF, and Save the Children to name a few—already manage data in humanitarian emergencies, the data available now are quantitatively and qualitatively different. This is due in part to the second characteristic, which is that the data are not generated exclusively by academics or professionals but by an enormous range of individuals, many of whom (e.g. users of mobile phones after the Haiti earthquake) may not even realize they are generating data. Third, the data on the ground are generated by technologies that are familiar, ubiquitous, and easy to use – most often a phone, increasingly an internet-enabled smartphone. One interesting theme emerging from many of these innovations is how much they are driven from the ground up, by users in the immediate or long-term aftermath of emergencies.

Many groups and organizations are doing innovative work in this area to generate and manipulate data. These groups range from small self-organized sets of individuals to intergovernmental organizations such as the ITU, an agency of the UN. Last year the Harvard Humanitarian Initiative (http://hhi.harvard.edu/), which draws together a number of related initiatives, released a report that discussed lessons learned from the use of data following the Haiti earthquake [2]. The key messages acknowledge the difficulties and cautioned that there is now an urgent need to rethink how humanitarian systems manage information. It recognized five key areas of specific need : (1) a neutral forum where agreement and conflict resolution could happen between the international humanitarian system and the volunteer and technical communities (V&TCs); (2) a way to allow innovation itself to happen; (3) a deployable field team with a mandate to implement the best available tools and practices from the V&TCs in the field; (4) a research and training consortium to evaluate the work in the field and to train humanitarians and V&TCs alike; and (5) an interface that outlines ways of collaborating before and during emergencies, with agreed-upon procedures for communication.

At its core the concept of digital humanitarianism is about better communication, and this of course is an ancient need. What is new are the possibilities offered by synthesizing these now-huge volumes of data. So where do journals fit in? In many ways these field data are reminiscent of the data that began to emerge in biology as sequencing accelerated along with associated technologies such as microarray and proteomics. In a famous plea in the form of a letter called “Show Me the Data” [3], a reviewer lamented the difficulty of assessing the rigor of the studies because of the lack of availability of the primary data. Later on, however, when such data were more available, the enormity of the task of reviewing papers based on these datasets became more obvious – and one reviewer then lamented in a review report that to adequately review one particular paper based on large amount of microarray data would, he estimated, take about the time that the PhD student whose paper it was had required to perform the experiments in the first instance – i.e., months, if not years. Such a realization begs the question of what peer review means in the context of such large datasets, and how can it be meaningfully presented – in the context of an academic journal or elsewhere. It's not clear to us.

As the HHI report noted, “the 2010 Haiti earthquake response will be remembered as the moment when the level of access to mobile and online communication enabled a kind of collective intelligence to emerge.” There is already much expertise and will to use these data, and, by being open source, most developers have made a commitment to enabling collaboration and reuse. We would argue there are a few key points that could enhance these innovations even more. Organizations working in this area should be encouraged to collaborate and disseminate their methods and findings, and perhaps dissemination of methodology is where journals can help most. First, minimum data and (even more crucially) metadata standards that can be applied across a range of settings should be rapidly developed and tested. Journals also may be able to help develop standards around privacy and storage of these data. Second, methods for storing and accessing the data need to be agreed upon so that the data can be reanalyzed as needed. Third, methods for analyzing these data need to be collected together so that different studies can be compared. But overall, there is a need for innovation to continue apace, and journals should work to facilitate the documentation of this innovation, not hinder it.

The rise of big datasets and their new uses being pioneered in humanitarian settings are exciting and innovative – and will only develop more. There are big challenges ahead to capture and harness the data and to ready the methodologies for the next time they are needed.

## Abbreviations

MSF: Médecins Sans Frontières
V&TC: volunteer and technical community
